# Functional Brain Connectivity as a New Feature for P300 Speller

**DOI:** 10.1371/journal.pone.0146282

**Published:** 2016-01-11

**Authors:** Aya Kabbara, Mohamad Khalil, Wassim El-Falou, Hassan Eid, Mahmoud Hassan

**Affiliations:** 1 Department of electrical and computer engineering, ULFG1, Tripoli, Lebanon; 2 Azm center for research in biotechnology and its applications, EDST, Tripoli, Lebanon; 3 Mazloum Hospital, Tripoli, Lebanon; 4 INSERM, U1099, F-35000, Rennes, France; 5 Université de Rennes 1, LTSI, F-35000, Rennes, France; Universiteit Gent, BELGIUM

## Abstract

The brain is a large-scale complex network often referred to as the “connectome”. Cognitive functions and information processing are mainly based on the interactions between distant brain regions. However, most of the ‘feature extraction’ methods used in the context of Brain Computer Interface (BCI) ignored the possible functional relationships between different signals recorded from distinct brain areas. In this paper, the functional connectivity quantified by the phase locking value (PLV) was introduced to characterize the evoked responses (ERPs) obtained in the case of target and non-targets visual stimuli. We also tested the possibility of using the functional connectivity in the context of ‘P300 speller’. The proposed approach was compared to the well-known methods proposed in the state of the art of “P300 Speller”, mainly the peak picking, the area, time/frequency based features, the xDAWN spatial filtering and the stepwise linear discriminant analysis (SWLDA). The electroencephalographic (EEG) signals recorded from ten subjects were analyzed offline. The results indicated that phase synchrony offers relevant information for the classification in a P300 speller. High synchronization between the brain regions was clearly observed during target trials, although no significant synchronization was detected for a non-target trial. The results showed also that phase synchrony provides higher performance than some existing methods for letter classification in a P300 speller principally when large number of trials is available. Finally, we tested the possible combination of both approaches (classical features and phase synchrony). Our findings showed an overall improvement of the performance of the P300-speller when using Peak picking, the area and frequency based features. Similar performances were obtained compared to xDAWN and SWLDA when using large number of trials.

## Introduction

A Brain Computer Interface (BCI) is a communication system in which messages or commands that an individual sends to the external world do not pass through the brain’s normal output pathways of peripheral nerves and muscles [1]. One of the most popular non-invasive BCIs is the so called 'P300 speller' that helps people suffering from severe neuromuscular disorders like amyotrophic lateral sclerosis (ALS), multiple sclerosis, cerebral palsy, spinal cord injuries and stroke. These disorders usually result in loss of voluntary muscle control due to the destruction of motor neurons with intact cognitive abilities [2][3]. Therefore, their only way to communicate with the environment is using brain activities.

A typical pipeline for a BCI involves several steps: the first one is the recording of brain activity using either invasive [4] or non-invasive recording system [1, 5–6]. The second step involves the pre-processing of the recorded data. This step is essentially realized to remove artifacts or non-useful information. The pre-processing step is followed by a feature extraction procedure such as time, frequency, time-frequency or spatial domain features. Finally, the extracted features are then processed for classification purpose and can be translated into actions [7]. To date, the most used features in P300 systems are derived from single channels, such as amplitude variations [8], calculation of area [7], matched filter [9], peak picking [7] and template-based cross correlation method [10]. In addition, more advanced techniques have been reported including stepwise linear discriminant analysis (SWLDA) [11][7], support vector machines [12][13][14], spatial filtering using xDAWN algorithm [15] and information geometry framework [16].

On the other side, it became evident to consider that the brain is a large-scale complex network [17]. Cognition as well as brain disorders were reported to be network phenomena [18–20]. Several studies have demonstrated that brain functions require the integration of distributed brain areas [21–25].

In this context, the functional connectivity approach has been used for motor imagery and has provided encouraging results for the classification of mental tasks in the context of BCIs [26–30]. Concerning the P300 Speller, a ‘qualitative’ MEG-fMRI based analysis have been recently reported in [31] using the coherence function. Authors showed that the coherent activity in the bilateral parieto-occipital cortices could play a significant role in the P300-BCI. However, an appropriate approach using functional connectivity in an economical P300 speller based on scalp electroencephalographic (EEG) signals has never been established yet, which is the objective of the presented paper.

Here, we propose a novel way to analyze the P300 waves obtained for target and non-targets cases. The new approach is based on the phase synchronization between the scalp EEG signals. The method was applied on ten subjects performing a task of recognizing Arabic letters presented on a screen. Results were compared with the widely used features and classification techniques. Finally, the possible combination of the existed approaches with the phase synchrony was also investigated.

## Materials and Methods

### 1. Data acquisition and experimental setup

EEG signals were recorded at MAZLOUM hospital (Tripoli, Lebanon) using a 19-channel EEG system (The NicoletOne^™^ Neurodiagnostic system). Electrodes were placed in accordance with the revised international 10/20 system [32] at Fp1, Fp2, F3, F4, F7, F8, C3, C4, P3, P4, O1, O2, T3, T4, T5, T6, Fz, Pz and Cz. Electrode impedance was always kept under 5 kΩ. The ground electrode was placed at the center of the forehead. The EEGs were sampled at 500 Hz, bandpass filtered at 0.1–80 Hz, and stored in EDF format.

**Fig 1:**
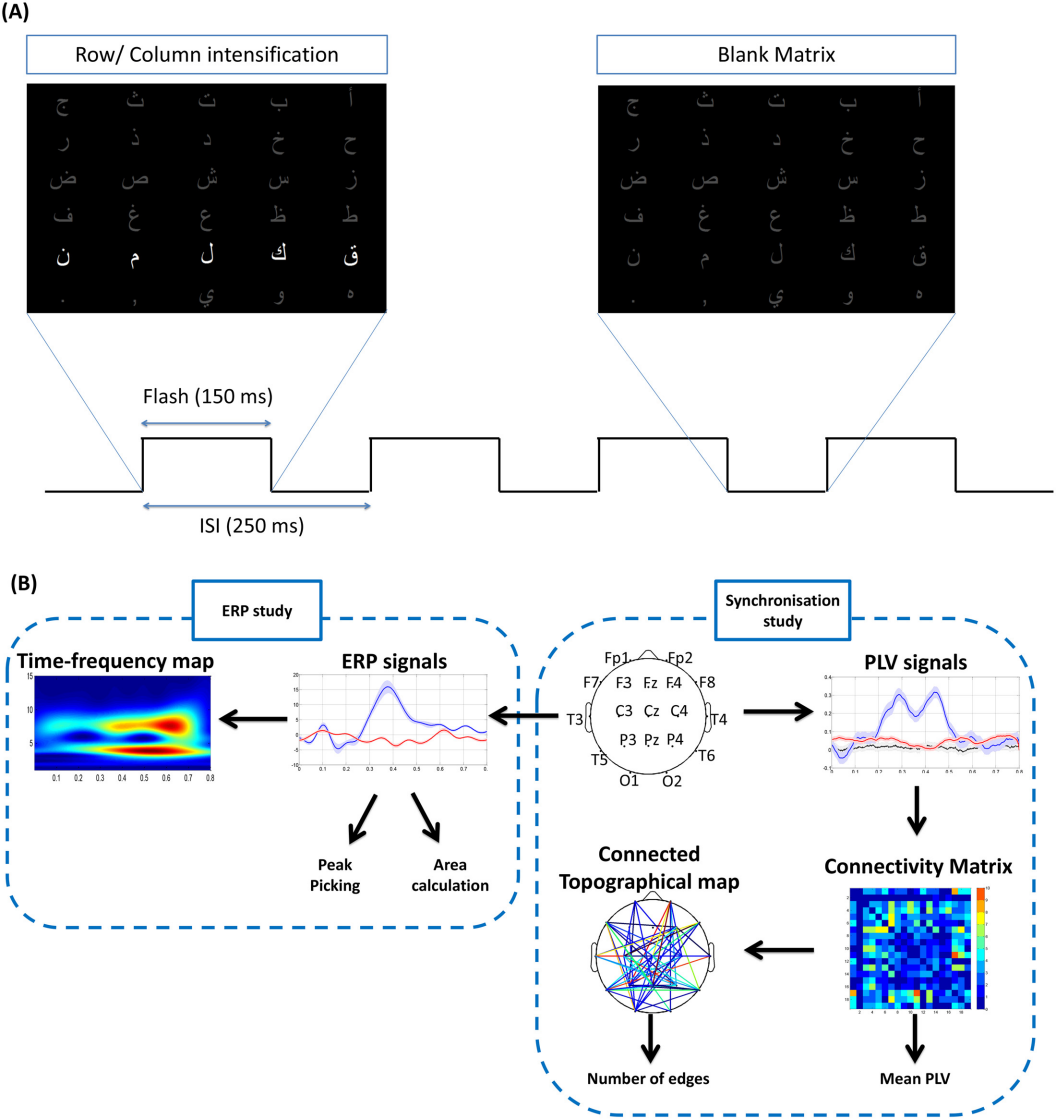
Paradigm design and the steps of analysis. (A) Paradigm design: The Arabic alphabets were presented on a screen. The time course of events was about 250 ms ISI. 11 stimuli (6 rows and 5 columns) were intensified in a random order for 150 ms. (B) The different steps of the phase synchronization and the ERPs study.

Ten right-handed subjects (5 males and 5 females) aged between 20 and 30 were participated in the experiment. None of them had any neurological deficits. This study was approved by the Local Ethics Committee at Mazloum Hospital, Lebanon. Subjects provided written informed consent before their participation. Participant was sitting in a comfortable chair in front of a computer monitor (344 x 194 mm) located at 60 cm away from his eyes. Participant was supposed not to have any muscle movements during the EEG recording. During the experiment, a 5x6 matrix containing Arabic letters (Fig 1) was presented on the computer screen. In a random order, each row or column was intensified for a short period of time ("Flash duration"). At each run, the participant was asked to focus on a specified letter of the matrix, and count silently the number of times of the flashes of this letter. Flashes of row or column containing the desired symbol constituted target stimuli and suppose to evoke a P300 wave, while all other flashes of rows and columns constituted non-target stimuli and may not evoke a P300. The P300 wave can be found theoretically in exactly one row and one column and their intersection was considered as the selected stimulus. This paradigm was adopted from [7].

In our system, row/column intensifications were randomized in blocks of 11 (5 row and 6 columns). Sets of 11 intensifications were repeated 15 times for each character. This repetition was necessary in order to reduce the measurement's noise recorded for each row and column's flash. Based on [7], the flash duration was equal to 150 ms, and the brief pause between two flashes was defined as inter-stimulus interval (ISI) and equal to 250 ms, as shown in Fig 1. A Matlab-based (MATLAB R2012, MathWorks) Graphical User Interface (GUI) was developed for presenting the stimuli.

Four sessions were realized for each subject. The first and the second sessions consisted of a series of separated characters. The third and the fourth sessions consisted of words. The sessions were separated by a break of 5–10 min. Each session contained 14 runs. After each run, the matrix was blank for 3s. This period informed the user that the previous character was completed and to focus on the next character that was displayed on the left of the screen. Thus, the dataset for each subject contained four raw signals in total. A total of 420 target trials (14 characters * 15 * 2) and 1890 non-target trials (14 characters * 15 * 9) was obtained.

### 2. Pre-processing

We extracted a window of length 1 s ranging from −200 ms before flash stimuli to 800 ms after flash stimuli (500 samples per trial). All EEG signals were filtered using FIR band-pass filter with 1–12 Hz cut-off frequencies [9]. This temporal filter was also performed to remove various undesired signals such as slow variations of the EEG signal (due to electrode polarization) or power-line interference (50 Hz in Lebanon). Moreover, eye blinks artifacts were rejected using an automatic method based on the Independent component analysis (ICA). The algorithm consisted of decomposing the raw EEG signals into 19 independent components (ICs), then removing the artifactual components automatically, based on the ratio between the weights of frontal sensors and those of other sensors [33].

### 3. Feature extraction

#### 3.1. Time domain features

To extract the P300 wave from EEG signals, we first compute the time-locked average across trials of the same type. All trials of a given type were aligned to a trigger that indicates the onset of the stimulus and then averaged over all trials and electrodes. A normalization step with respect to the baseline window was then performed. To investigate the changes of event-related potentials in brain activity before and after the stimulus, we used the pre-stimulus period as a baseline [-200 0] ms and perform a "z-score approach" normalization on the window [0 800 ms] already defined. This approach was adopted as it supposed to take into account the variability of the baseline values [34].

Two features were extracted from the ERPs:

- Peak picking feature [7]: Peak picking represents the difference between the lowest negative point prior to the P300 window (the window for the P300 ranged typically between 220 and 500 ms) and the highest positive point in the P300 window.
- Area feature [7]: This feature represents the sum of the data points present in the P300 window.

#### 3.2. Time-frequency features

Time-frequency EEG analyses may provide additional information about dynamical changes of the electrical activity, not apparent in the temporal signal [35]. In the present study, continuous wavelet transform (CWT) was used to compute the time-frequency maps of the normalized ERP signals. A wavelet is a small wave that has an oscillating wavelike characteristic and has its energy concentrated in time [36]. The definition of continuous wavelet transform is given by Eq (1):

$$\text{X}(\text{a},\text{b})=\frac{1}{\sqrt{\text{b}}}\int_{-\infty }^{\infty }\text{x}(\text{t})\Psi \left(\frac{\text{t}-\text{a}}{\text{b}}\right)\text{dt}$$ (1)

where *x*(t) is the analyzed signal, a shifts time, b (>0) modulates the width, and Ψ (t) is a mother wavelet.

In this paper, we used the complex Morlet wavelet as it is most commonly used in the EEG time-frequency analysis, defined as follows [37]:

$$\Psi \left(\text{t}\right)=\frac{1}{\sqrt{{\pi \text{f}}_{\text{b}}}}{\text{e}}^{\text{i}2{\pi \text{f}}_{\text{c}}\text{t}}{\text{e}}^{-\frac{{\text{t}}^{2}}{{\text{f}}_{\text{b}}}}$$ (2)

where f_b_ is the bandwidth parameter, f_c_ is the wavelet center frequency, and p is an integer parameter.

The time-frequency transform was computed in each trial and then averaged over all trials and electrodes.

#### 3.3. Proposed feature: Phase synchronization

Phase synchronization (PS) was used here as it provides an amplitude-free measure of connectivity between cortical regions. PS was shown to be less susceptible to the effects of artifacts and inter-trial / inter-subject amplitude variability [38]. The first step for estimating the phase synchrony is to extract the instantaneous phase of each signal. We used the method based on Hilbert transform in our study. The second step is the definition of an appropriate index to measure the degree of synchronization between estimated instantaneous phases. To measure phase synchrony, the phase locking value (PLV) method was used as described in [39]. For each source pair, *x* and *y*, at time *t (t* = *t*_*1*_,…, *t*_*T*_ where *T* = *D* * *f*_s_; *D* and *f*_s_ denote the signal length relative to the onset and the sampling frequency, respectively) for the *Tr* trials and for subject *k* (*k* = 1…*M*, where *M* denotes the number of subjects), PLV is defined as:

$$PLV_{xy}^{k}\left(t\right)=\frac{1}{Tr}\left|\sum_{n=1}^{N}exp\left(\text{j}(\Phi_{x}\left(t\right)-\Phi_{y}\left(t\right)\right))\right|$$ (3)

In our study, the data was recorded from 19 EEG electrodes, this implies 171 possible instantaneous PLV signal for every condition. PLV time courses were computed over multiple trials of a same stimulus, and the transient changes in connectivity were observed in the pre-defined time window of analysis.

A normalization process was also applied to ERP signals by considering the baseline [-200 0] ms and response [0 800] ms windows.

To statistically validate the significance of the identified networks, we compared them with PLVs computed from surrogate data [40]. A set of 200 surrogate time series was generated by randomizing the phase of the original signals. We only retained results when the difference between PLVs computed on the surrogate data and PLVs computed on the original time series was significant (*p<0*.*01*).

To obtain the connectivity matrix, the instantaneous PLV signals were then averaged within a time window. Selecting the time window was a delicate part. Based on the facts that the evoked potentials appear about 300 ms after the stimulus, we chose the [200 500] ms time window.

In order to visualize the PLV values between electrodes, topographical connected maps were illustrated. We were interested in displaying only the significant edges retained from the surrogates test. A statistical analysis was performed using the Wilcoxon test, in order to quantify the differences between target and non-target stimulus, in the ERPs, PLVs and TF maps.

### 4. Classification methods

After extracting the number of edges of the topographical maps as a feature (Fig 1), data were classified as ‘target’ or ‘non-target’ responses using a support vector machine classifier. We used different methods existing in the state-of-the art of “P300 speller” to compare them with the proposed method:

- xDAWN algorithm [15]: xDAWN is a spatial filtering algorithm based on SVD and PCA. It attempts to define an estimation of the subspace that contains P300 evoked potentials. Then a Bayesian Linear Discriminant Analysis (BLDA) is used to classify epochs as either ‘target’ or ‘non-target’.
- Stepwise linear discriminant analysis (SWLDA) [11]: The ability of SWLDA to provide good results in classifying P300 waves has been confirmed in [41]. The stepwise LDA applies a regular LDA after reducing the feature space by selecting suitable features to be included in the discriminant function.
- Peak Picking algorithm: After extracting the peak picking feature of the ERP signals, a linear support vector machine (SVM) classifier was used for classification.
- Area algorithm: A SVM classifier was used for classification after extracting the area feature.

In order to evaluate the classification performance of each of the algorithm, the following steps were realized: (a) A feature vector was built from the large set of N_total_ ERP signals generated from the four raw EEG signals of each subject (as mentioned in Section 1), (b) Data were divided into training/testing sets using the cross-validation procedure to prevent over-fitting, (c) The classifier was trained using the training data set(d) The classifier was then applied to the rest of the N_test_ characters and the accuracy values were calculated, the (b)-(c)-(d) processes were repeated 10 times, and finally (e) the accuracy was defined as the average of the accuracies calculated over the repetitions. To analyze the statistical differences in the performances obtained from the different classification algorithms, the Wilcoxon rank-sum test (*p<0*.*05*) was used.

## Results

**Fig 2:**
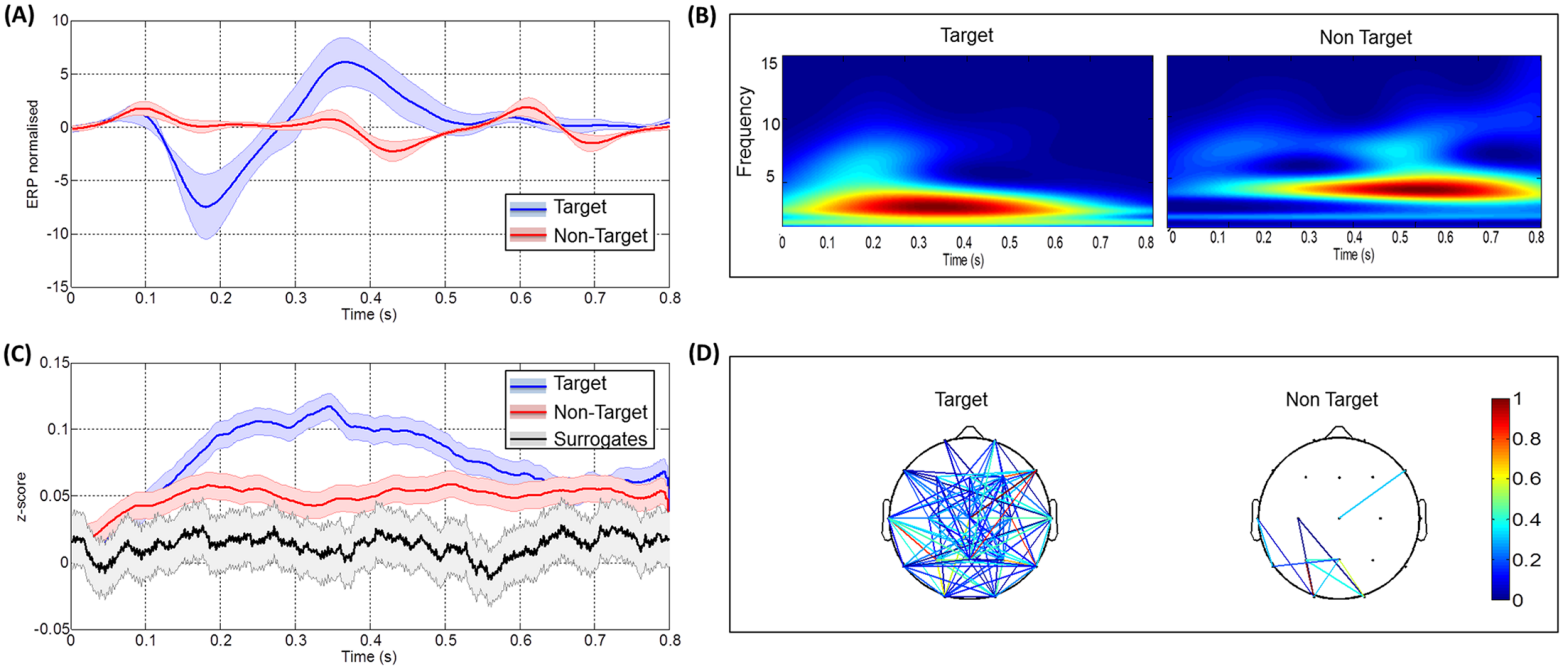
Results obtained after averaging over all trials, electrodes and subjects. (A) The target and non-target ERP responses, (B) The two frequency maps corresponding to both conditions, (C) The target, non-target PLV responses. The black line represents the phase synchrony computed on surrogate data and the grey strip indicates dispersion of these data ± standard deviation and (D) The two connected topographical scalps for target and non-target responses.

First, we present the results obtained after measuring the synchronization on a very large number of trials, extracted from all sessions (420 target trials and 1890 non-target trials). The PLVs and ERP signals were averaged over all subjects. Fig 2 shows the differences between the evoked related potentials of target and non-target conditions (Fig 2A), the corresponding time-frequency maps (Fig 2B) and the phase synchrony (Fig 2C). In Fig 2C, a clear difference between the target and non-target was found in the time range between 100 ms and 650 ms after stimuli. The Fig 2D shows the PLV connectivity maps obtained for each condition.

**Fig 3:**
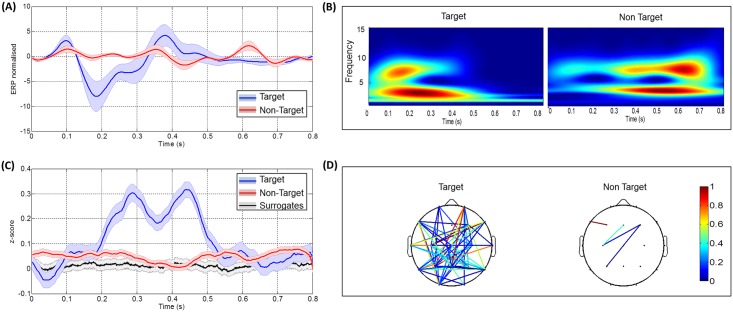
Results obtained for a given subject after averaging over all trials and electrodes. (A) The target and non-target ERP responses. (B) The two frequency maps corresponding to each condition. (C) The target, non-target PLV responses. The black line represents the phase synchrony computed on surrogate data and the grey strip indicates dispersion of these data ± standard deviation and (D) The two connected topographical scalps for target and non-target responses.

In Fig 3, a typical example of the results obtained for a given subject is reported (see S1, S2, S3, S4 and S5 Figs for more examples). In both analyses, a statistical difference between target and non-target was reported in the case of ERPs (*p*<0.05) and PLVs (*p*<0.005). However, no significant difference between the two conditions was detected in the time-frequency analysis (*p* = 0.34 and *p* = 0.5 in Figs 2 and 3 respectively). After the comparison with surrogates data, a large number of edges were remained in the connected topographical map in the case of target trials (left), while a very few connected edges were conserved in the case of non-target trials (right).

**Fig 4:**
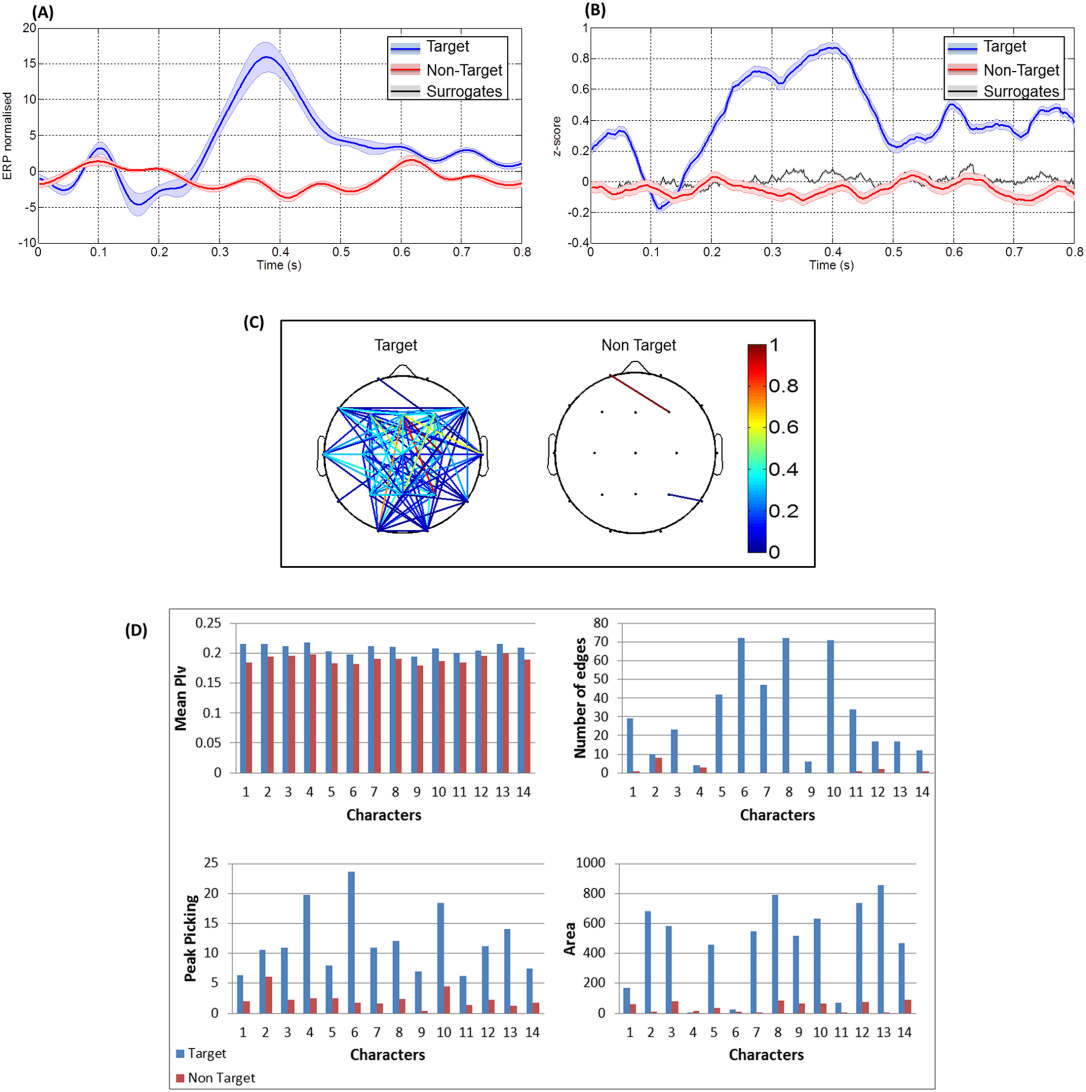
Results obtained from subject 1, after averaging the trials corresponding to 14 characters. (A) The target and non-target ERP responses, (B) The target, non-target PLV responses. The black line represents the phase synchrony computed on surrogate data and the grey strip indicates dispersion of these data ± standard deviation. (C) The two connected topographical scalps for target and non-target responses and (D) Comparison between the different features extracted with respect to the 14 characters.

**Fig 5:**
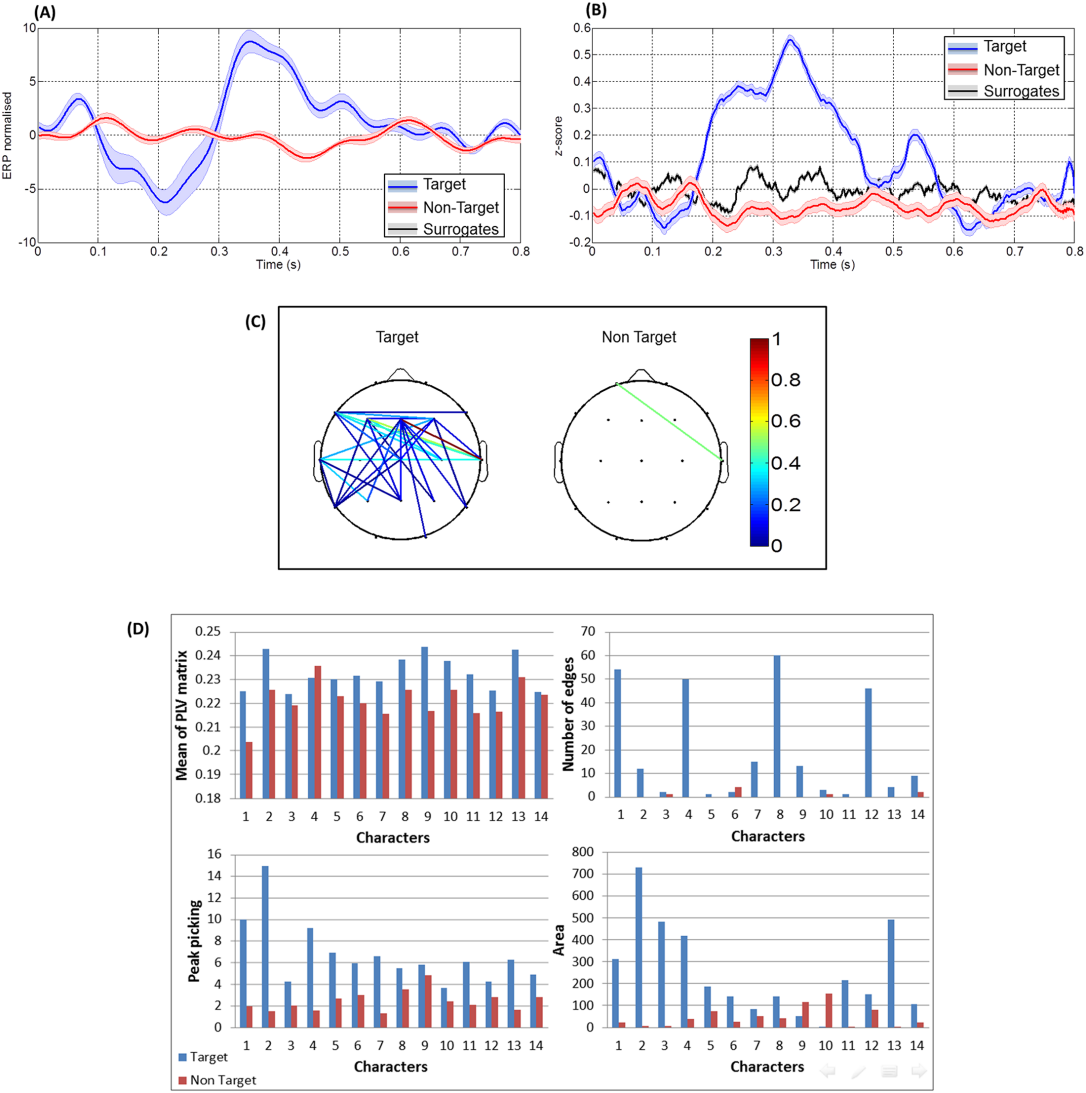
Results obtained from subject 2, after averaging the trials corresponding to 14 characters. (A) shows the target and non-target ERP responses. (B) shows the target, non-target PLV responses. The black line represents the phase synchrony computed on surrogate data and the grey strip indicates dispersion of these data ± standard deviation. (C) shows the two connected topographical scalps for target and non-target responses. (D) shows a comparison between the different features extracted with respect to the 14 characters.

In this section, we present the results obtained after reducing the number of trials to that corresponding to one character (the number of trials of target responses was 30 trials, and the number of trials of non-target responses was 135 trials). For each subject, PLVs and ERPs were averaged over all the characters per session (14 characters). Figs 4 and 5 show the results obtained for two different subjects. Figs 4A and 5A demonstrate the difference in ERP signals while Figs 4B and 5B show the difference in the PLVs. The different connectivity maps are shown in Figs 4C and 5C. The Fig 5D displays how the different extracted features differentiate between target and non-target trials. For the first subject, the mean PLV value was greater in the case of target than non-target condition. The chart at the top right of Fig 4D shows the presence of more edges in the target condition. Results obtained using the peak picking and the area features indicate similar results. Note that no error was detected in the case of mean PLV, the peak picking, and the number of edges features while 4 errors were observed in the case of the area feature.

**Fig 6:**
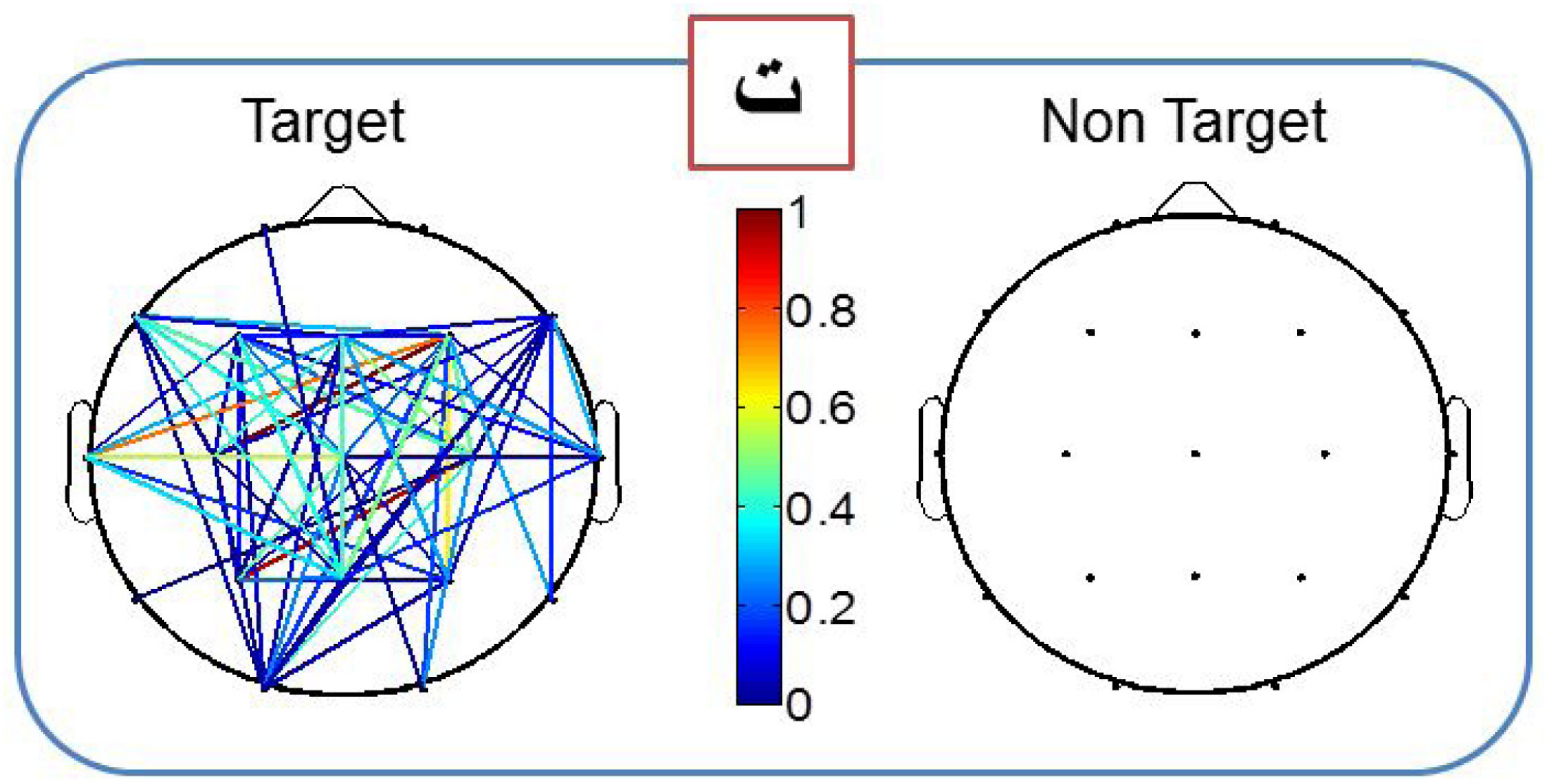
Results obtained after averaging all trials of target and non-target conditions for the character 'ت'.

For the second subject, an error in classification was detected with the mean PLV feature (character 4), and two errors were detected (character 3 and character 6) when counting the number of edges. No error was detected with the peak picking feature, and two errors were detected with the area feature (character 9 and character 10). In Fig 6, we illustrate the difference between the two connected map corresponding to target and non-target conditions for one character (see S6, S7, S8, S9 and S10 Figs for more examples).

**Fig 7:**
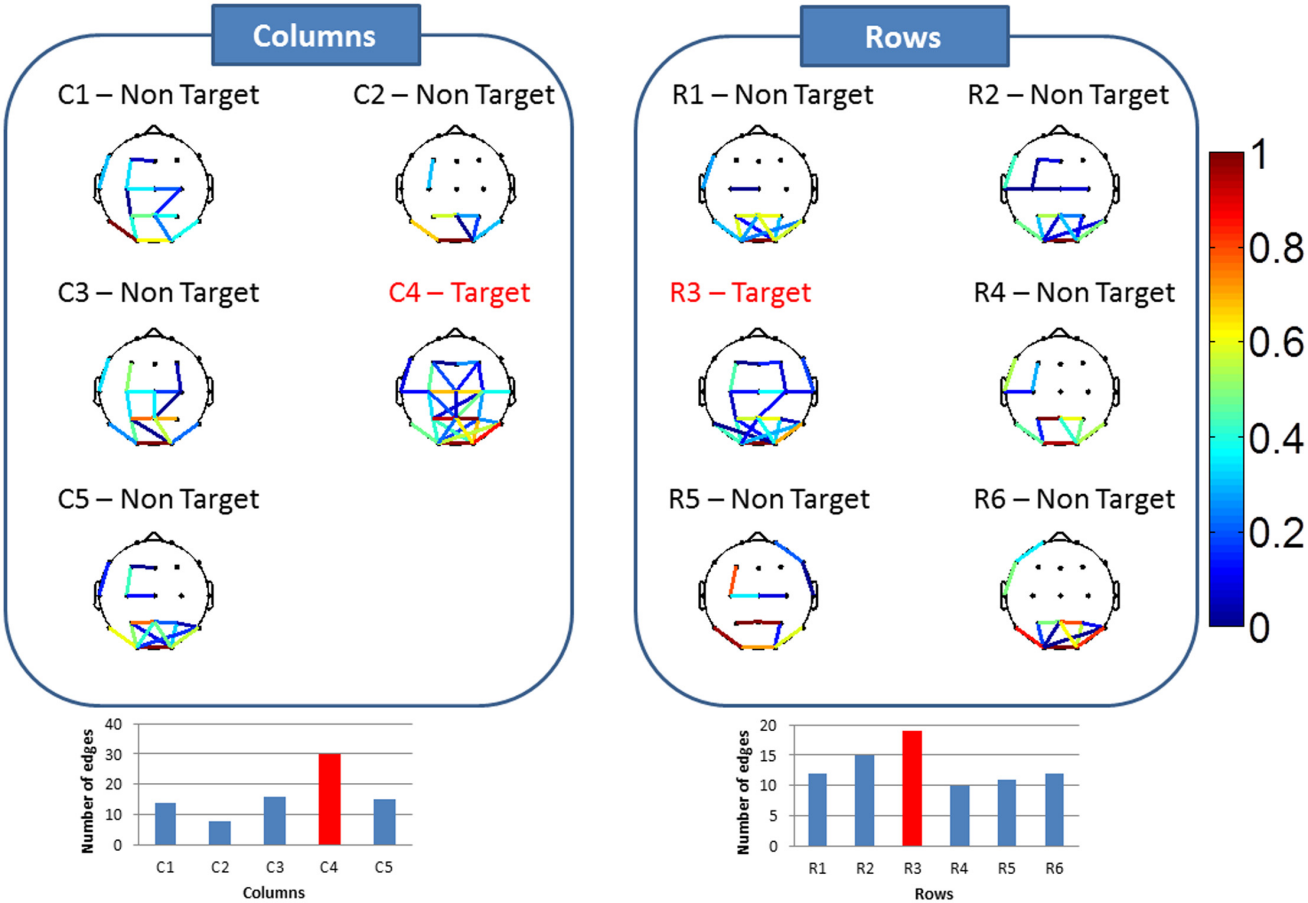
Typical example of results obtained after averaging all trials corresponding to each row/column response. The focused target character is from row 3, column 4: 'ص'.

We then realized the same process on the trials corresponding to one row/column intensification (15 trials). A typical example of the connectivity maps of the eleven stimuli is shown in Fig 7. We show that the number of edges corresponding to the target row (row 3 in this example) was the highest between all rows. Similarly, the number of edges corresponding to the target column (column 4 in this example) was the highest between all columns. By considering the intersection of the predicted row and the predicted column, we obtain for the given subject, 48 well-predicted letters of 56 letters.

**Fig 8:**
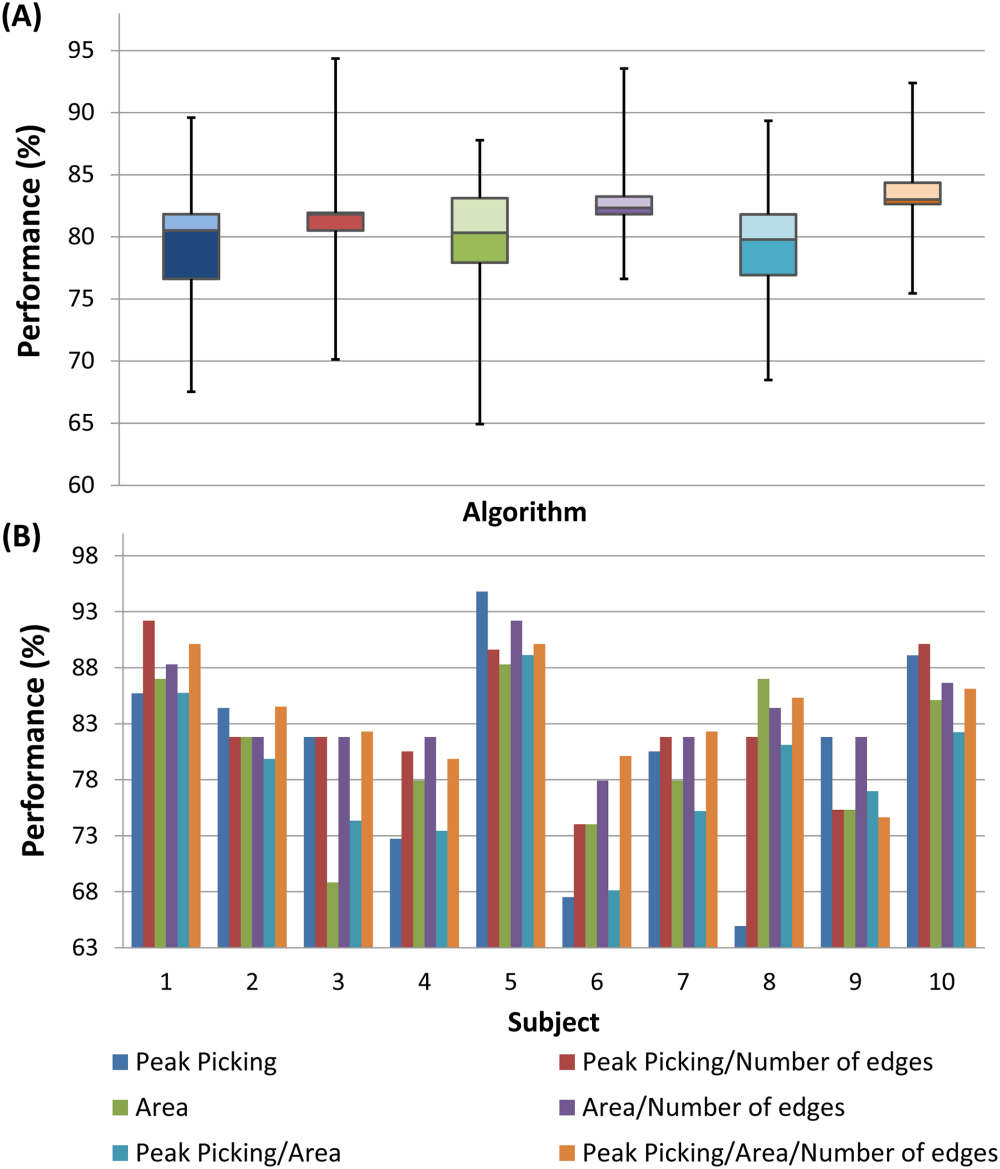
The performance of the different features applied solely or in combination with the phase synchrony. (A) Performance over participants (B) Performance for each participant.

Additionally, we have compared the performance of using only the classical features (the peak picking and the area) or combining them with the phase synchrony. Results are shown in Fig 8. The Fig 8A shows that the performance over subjects was improved when combining both approaches. Fig 8B shows the performance over each of the ten subjects. The phase synchrony dramatically improved the performance of the peak picking method on subjects 4 (72.72% vs. 80.52%), 6 (67.53% vs. 74.03%) and 8 (64.93% vs. 81.81%).

Results also showed that the combination of the two traditional features does not lead to better performance. However, a higher performance was obtained when combining the three features (peak amplitude, area and PLV).

**Fig 9:**
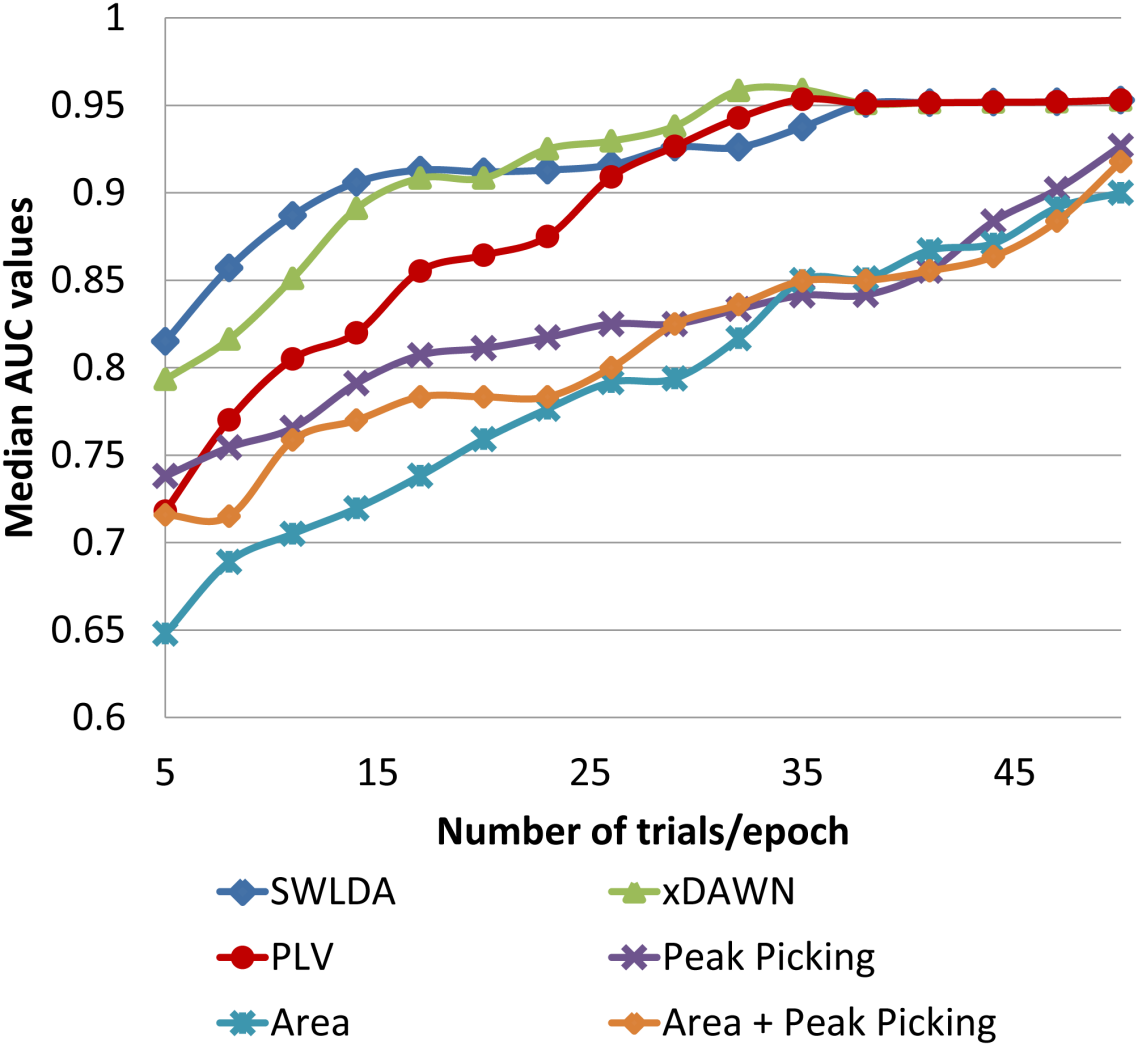
The performances of the different classification algorithms as a function of the number of trials.

Finally, the effect of the number of trials on the classification performance was evaluated in Fig 9. For each classification algorithm, we compute the Area Under ROC Curve (AUC) values in function of the number of trials. The illustrated AUC values represent the median values across all analyzed subjects. The Fig 9 reveals that increasing the number of trials lead to better performance accuracy for all the classification methods. One can notice also that the classification based on phase synchrony achieves better performance than methods based on peak picking or area features. The results of the Wilcoxon rank-sum test indicated a significant difference between the phase synchrony and the classification based on peak picking feature (*p*<0.01), area feature (*p*<0.01) and their combination (*p*<0.01). However, while our method provides better performance than SWLDA using 28 or more trials, it underperforms xDAWN and SWLDA algorithms for a small number of trials. The Wilcoxon test shows that there is a significant difference between xDAWN and PLV algorithms (*p*<0.01). No significant difference was observed between SWLDA and the phase synchrony.

## Discussion

In this paper, we used the functional connectivity analysis to characterize the evoked responses (ERPs) obtained for target and non-targets visual stimuli in the context of P300 speller. The functional connectivity was computed using the Phase Locking Value (PLV). The results obtained have demonstrated that the synchronization between different brain areas allows the classification of different type (target vs. non target) of visual stimuli. The results showed that the separation was also possible in case of 30 trials for the same condition. Compared to the classical features (mainly the peak picking and the area), the PLV provided globally better separation between target and non-target trials.

The results reported that the reduction of trials number has an important impact on the PLV performance. The results of PLV on 15 trials have provided good classification results for only 3 subjects. We then tested the possible combination of PLV with the peak picking and area.

The detection of target from a single trial was very challenging task in our application. Many recent researches have proposed different ways to reduce the number of trials while keeping a high accuracy rate. Some studies have proposed a boosting approach [42] or applying a complex classifier rather than a simple average based on applying an ensemble of SVM classifiers [12]. Another approach was presented in [16], where authors have classified a single trial ERP epoch, while reducing the number of repetitions needed for the training phase. Authors have also dealt with the problem of inter-session and inter-subject variability. Methodologically, PLV is measuring the ‘consistency’ in the phase difference between two signals, and consistency is measured across trials. Therefore, the application of PLV in single-trial context seems to be inadequate.

Here, our main objective was to introduce the functional connectivity as a new feature for the classification of visual evoked responses. The functional connectivity quantified by PLV revealed interesting results in characterizing both target and non-target evoked responses. We then investigated the feasibility of the functional connectivity in the P300-speller context. Results obtained by the phase synchrony showed higher performance than the classical features (such as peak picking and area). However, the phase synchrony method offered comparable results to the more advanced classification algorithms (SWLDA and xDAWN) when using a large number of trials.

Finally, we showed encouraging results about the use of the functional connectivity in the context of ERP analyses and P300 speller when using large number of trials. Nevertheless, we believe that several methodological issues should be tackled to achieve a good performance when applying the functional connectivity for a small number of trials in the context of a “P300 speller”. A possible direction in this context could be the reduction of trials number when computing functional connectivity [43] such as the so-called ‘single-trial’ connectivity methods. The dynamical behaviors of the functional networks can be also investigated to improve the classification results [44].

In addition, it is important to keep in mind that measuring the functional connectivity at the EEG scalp level may be affected by the problem of volume conduction. To address this problem, the phase slope index method suggested by [45] could be a good alternative. Moreover, the ‘EEG source connectivity’ analysis was shown to reduce the effect of the volume conduction and improve the spatial resolution of the networks [46]. We are, however, aware that methodological efforts are required to adapt these approaches in ‘real time’ perspective.

## Conclusion

In this paper, we showed that functional connectivity computed using the phase locking value is a very efficient tool to characterize visual evoked responses. Functional connectivity was applied on data recorded from subjects performing the task of recognizing Arabic letters presented on a screen, in the context of “P300 speller” paradigm. Results revealed a clear difference between functional networks obtained in the case of target and non-targets visual stimuli. In the context of the P300 speller, our findings reported that the phase synchrony is very powerful in the presence of a large number of trials. Other connectivity measures (mainly the single-trial methods) could be applied for faster and more efficient “P300 speller” system. We speculate that the proposed method can be easily extended to other Brain Computer Interface (BCI) systems.

## Supporting Information

S1 Fig Results obtained for subject 1, after averaging over all trials and electrodes. (A) The target and non-target ERP responses. (B) The two frequency maps corresponding to each condition. (C) The target, non-target PLV responses. The black line represents the phase synchrony computed on surrogate data and the grey strip indicates dispersion of these data ± standard deviation and (D) The two connectivity maps for target and non-target responses.
(TIF)

S2 Fig Results obtained for subject 2, after averaging over all trials and electrodes. (A) The target and non-target ERP responses. (B) The two frequency maps corresponding to each condition. (C) The target, non-target PLV responses. The black line represents the phase synchrony computed on surrogate data and the grey strip indicates dispersion of these data ± standard deviation and (D) The two connectivity maps for target and non-target responses.
(TIF)

S3 Fig Results obtained for subject 3, after averaging over all trials and electrodes. (A) The target and non-target ERP responses. (B) The two frequency maps corresponding to each condition. (C) The target, non-target PLV responses. The black line represents the phase synchrony computed on surrogate data and the grey strip indicates dispersion of these data ± standard deviation and (D) The two connectivity maps for target and non-target responses.
(TIF)

S4 Fig Results obtained for subject 4, after averaging over all trials and electrodes. (A) The target and non-target ERP responses. (B) The two frequency maps corresponding to each condition. (C) The target, non-target PLV responses. The black line represents the phase synchrony computed on surrogate data and the grey strip indicates dispersion of these data ± standard deviation and (D) The two connectivity maps for target and non-target responses.
(TIF)

S5 Fig Results obtained for subject 5, after averaging over all trials and electrodes. (A) The target and non-target ERP responses. (B) The two frequency maps corresponding to each condition. (C) The target, non-target PLV responses. The black line represents the phase synchrony computed on surrogate data and the grey strip indicates dispersion of these data ± standard deviation and (D) The two connectivity maps for target and non-target responses.
(TIF)

S6 Fig Results obtained after averaging all trials of target and non-target conditions for the character 'أ'. (TIF)

S7 Fig Results obtained after averaging all trials of target and non-target conditions for the character 'س'. (TIF)

S8 Fig Results obtained after averaging all trials of target and non-target conditions for the character 'ض'. (TIF)

S9 Fig Results obtained after averaging all trials of target and non-target conditions for the character 'ك'. (TIF)

S10 Fig Results obtained after averaging all trials of target and non-target conditions for the character 'م'. (TIF)

S11 Fig Results obtained after averaging all trials of target and non-target conditions for the character 'ف'. (TIF)
