# Supplementary figures and images for: Functional Brain Connectivity as a New Feature for P300 Speller
Source: PLoS One. 2016 Jan 11;11(1):e0146282. doi: 10.1371/journal.pone.0146282 (PMC4709183; doi:10.1371/journal.pone.0146282)

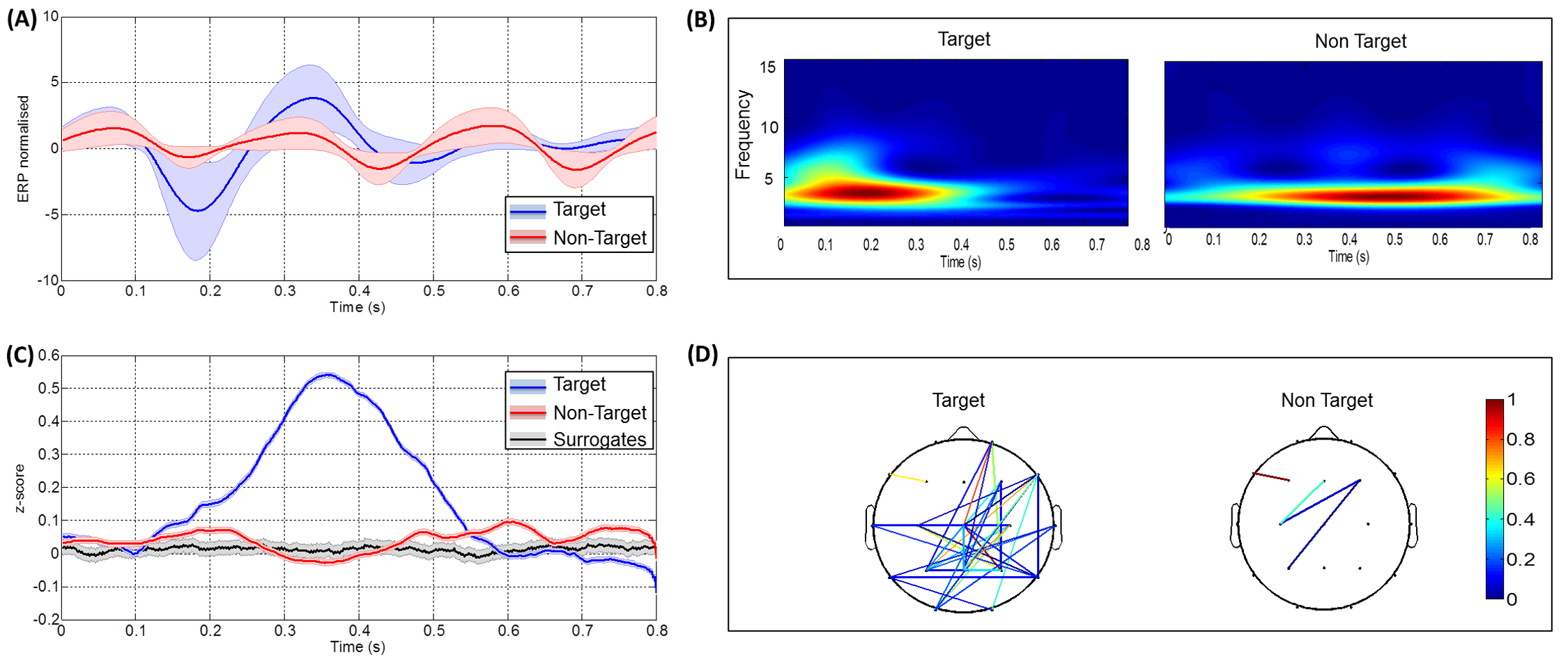

Supplement: S1 Fig — (A) The target and non-target ERP responses. (B) The two frequency maps corresponding to each condition. (C) The target, non-target PLV responses. The black line represents the phase synchrony computed on surrogate data and the grey strip indicates dispersion of these data ± standard deviation and (D) The two connectivity maps for target and non-target responses. (TIF) [file pone.0146282.s001.tif]

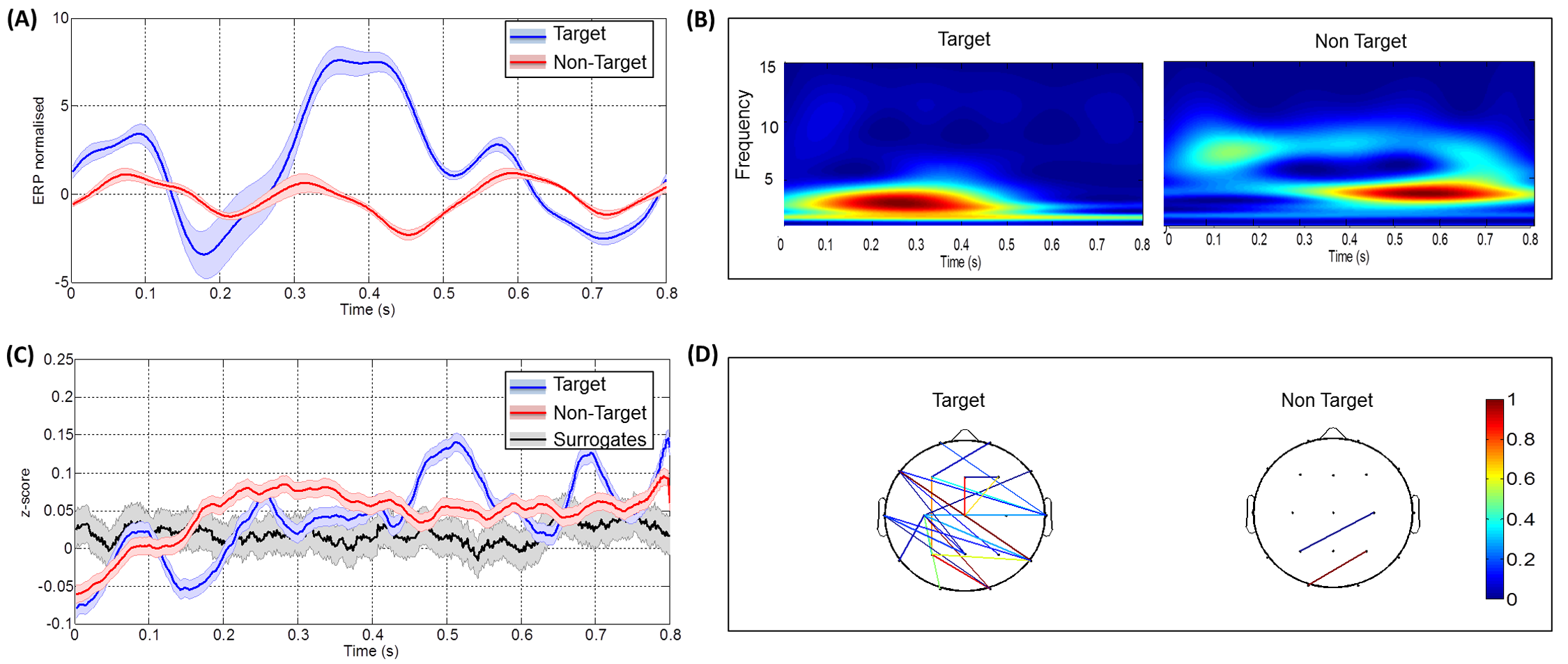

Supplement: S2 Fig — (A) The target and non-target ERP responses. (B) The two frequency maps corresponding to each condition. (C) The target, non-target PLV responses. The black line represents the phase synchrony computed on surrogate data and the grey strip indicates dispersion of these data ± standard deviation and (D) The two connectivity maps for target and non-target responses. (TIF) [file pone.0146282.s002.tif]

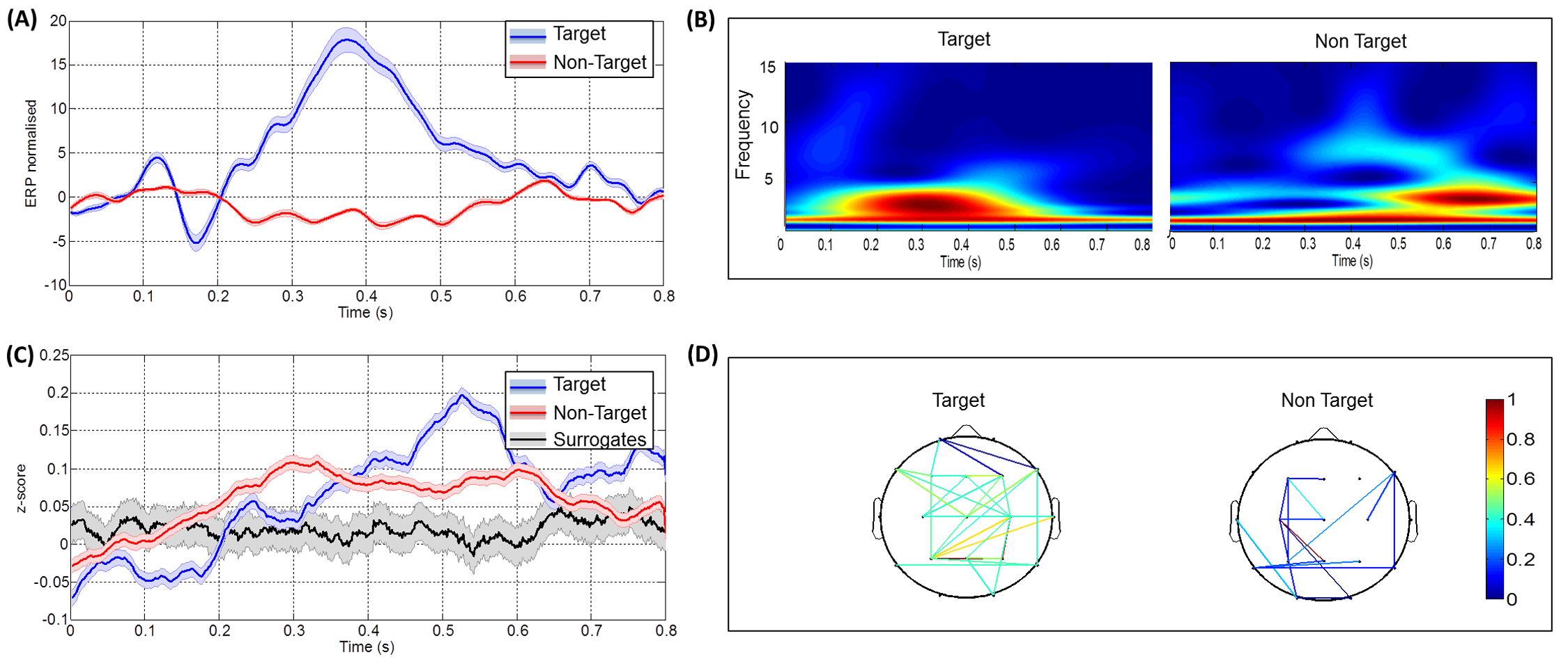

Supplement: S3 Fig — (A) The target and non-target ERP responses. (B) The two frequency maps corresponding to each condition. (C) The target, non-target PLV responses. The black line represents the phase synchrony computed on surrogate data and the grey strip indicates dispersion of these data ± standard deviation and (D) The two connectivity maps for target and non-target responses. (TIF) [file pone.0146282.s003.tif]

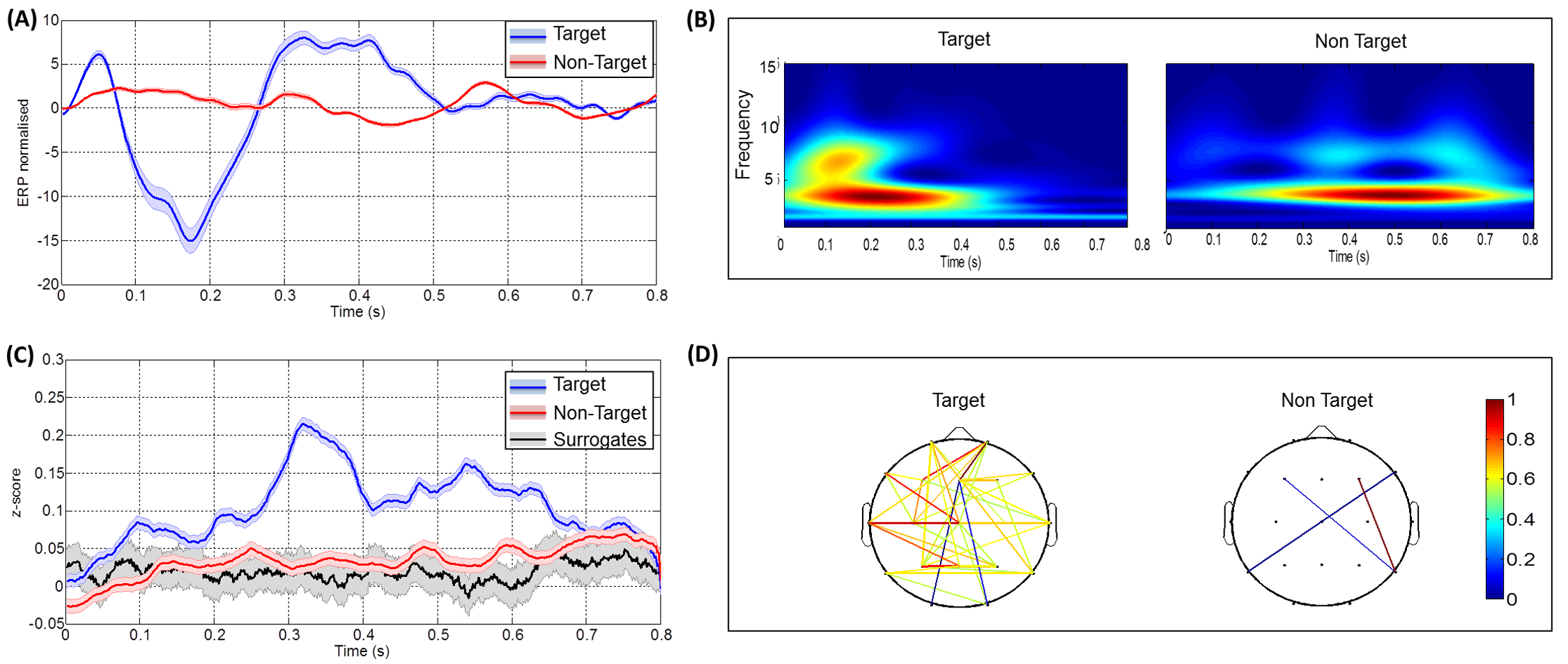

Supplement: S4 Fig — (A) The target and non-target ERP responses. (B) The two frequency maps corresponding to each condition. (C) The target, non-target PLV responses. The black line represents the phase synchrony computed on surrogate data and the grey strip indicates dispersion of these data ± standard deviation and (D) The two connectivity maps for target and non-target responses. (TIF) [file pone.0146282.s004.tif]

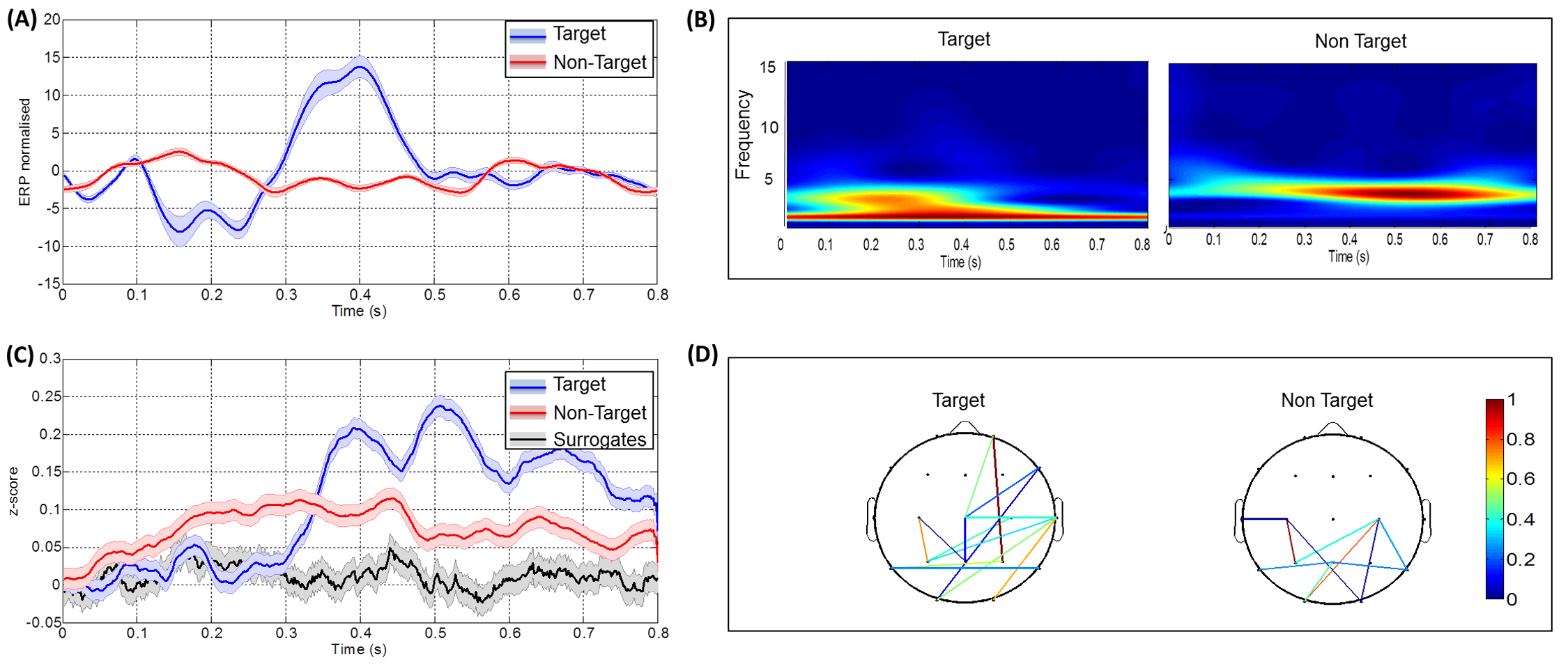

Supplement: S5 Fig — (A) The target and non-target ERP responses. (B) The two frequency maps corresponding to each condition. (C) The target, non-target PLV responses. The black line represents the phase synchrony computed on surrogate data and the grey strip indicates dispersion of these data ± standard deviation and (D) The two connectivity maps for target and non-target responses. (TIF) [file pone.0146282.s005.tif]

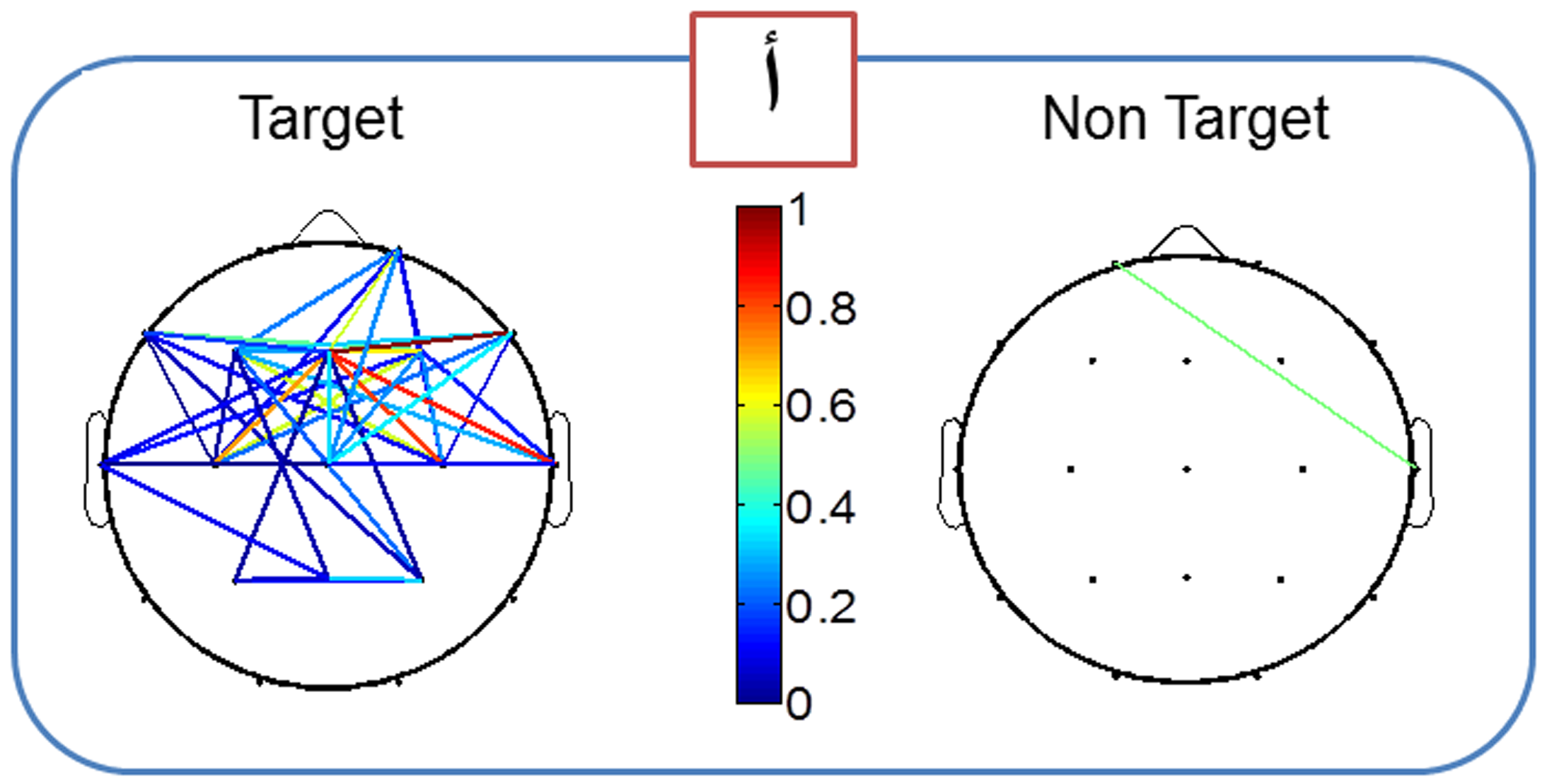

Supplement: S6 Fig — (TIF) [file pone.0146282.s006.tif]

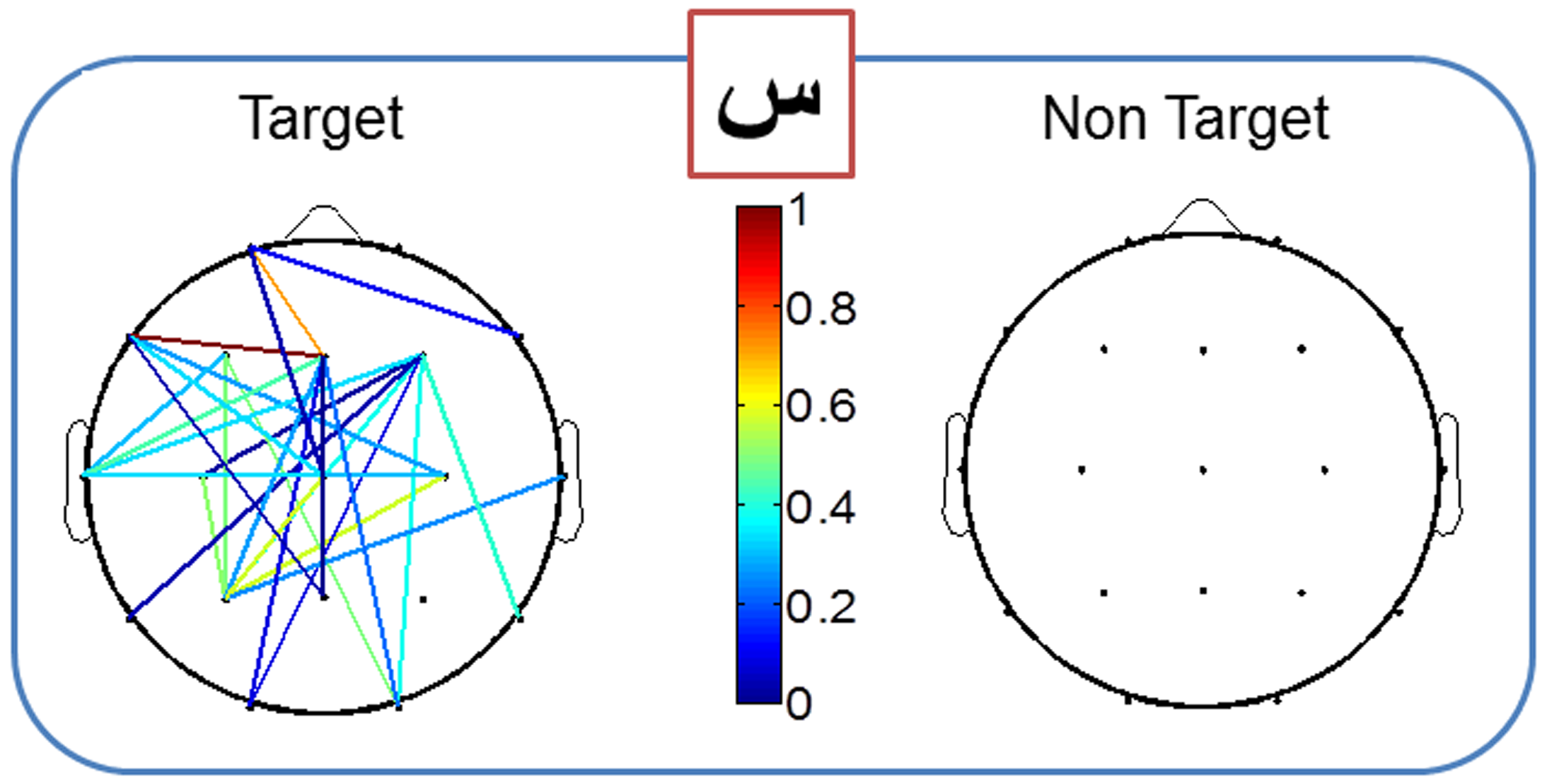

Supplement: S7 Fig — (TIF) [file pone.0146282.s007.tif]

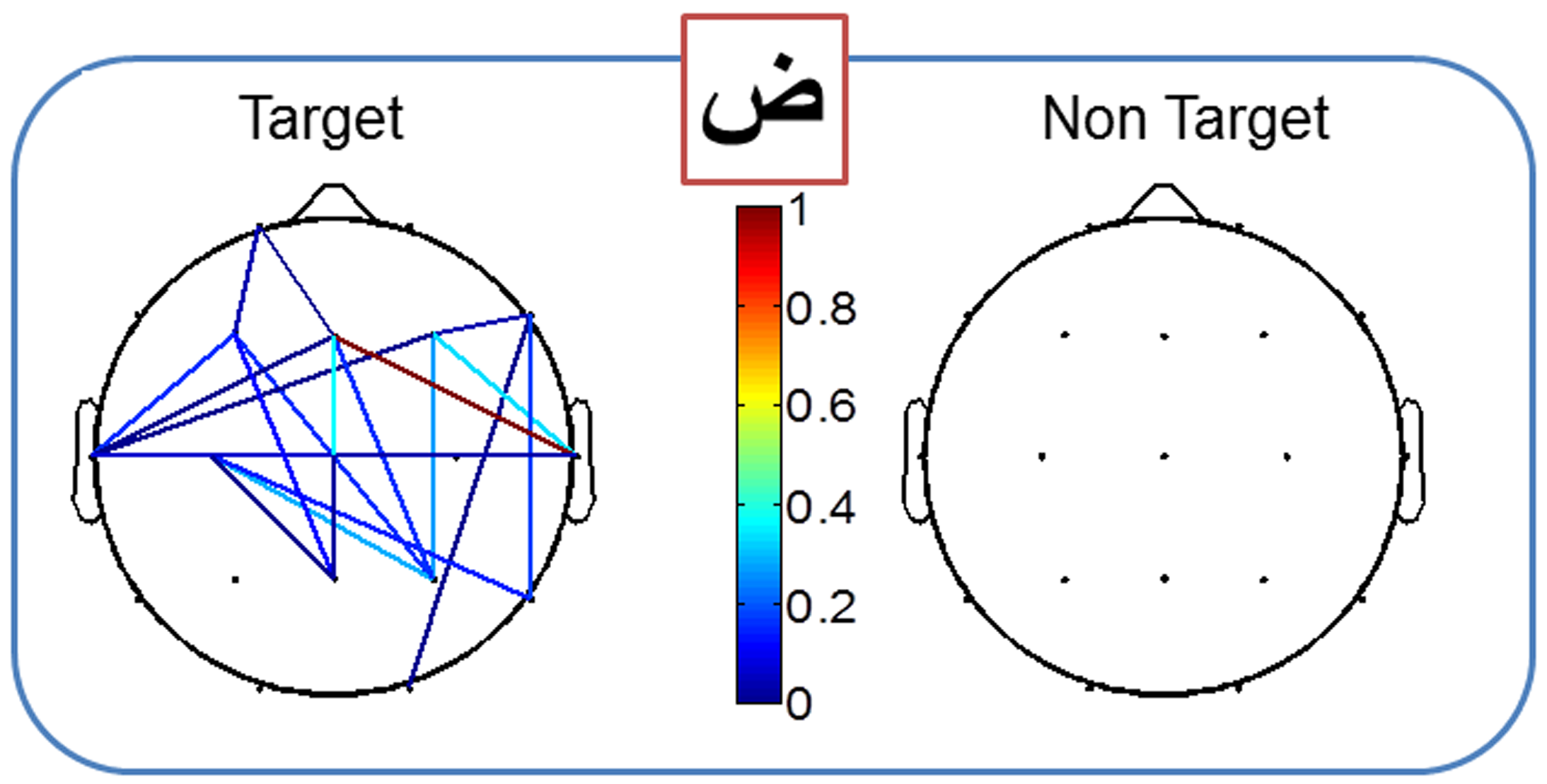

Supplement: S8 Fig — (TIF) [file pone.0146282.s008.tif]

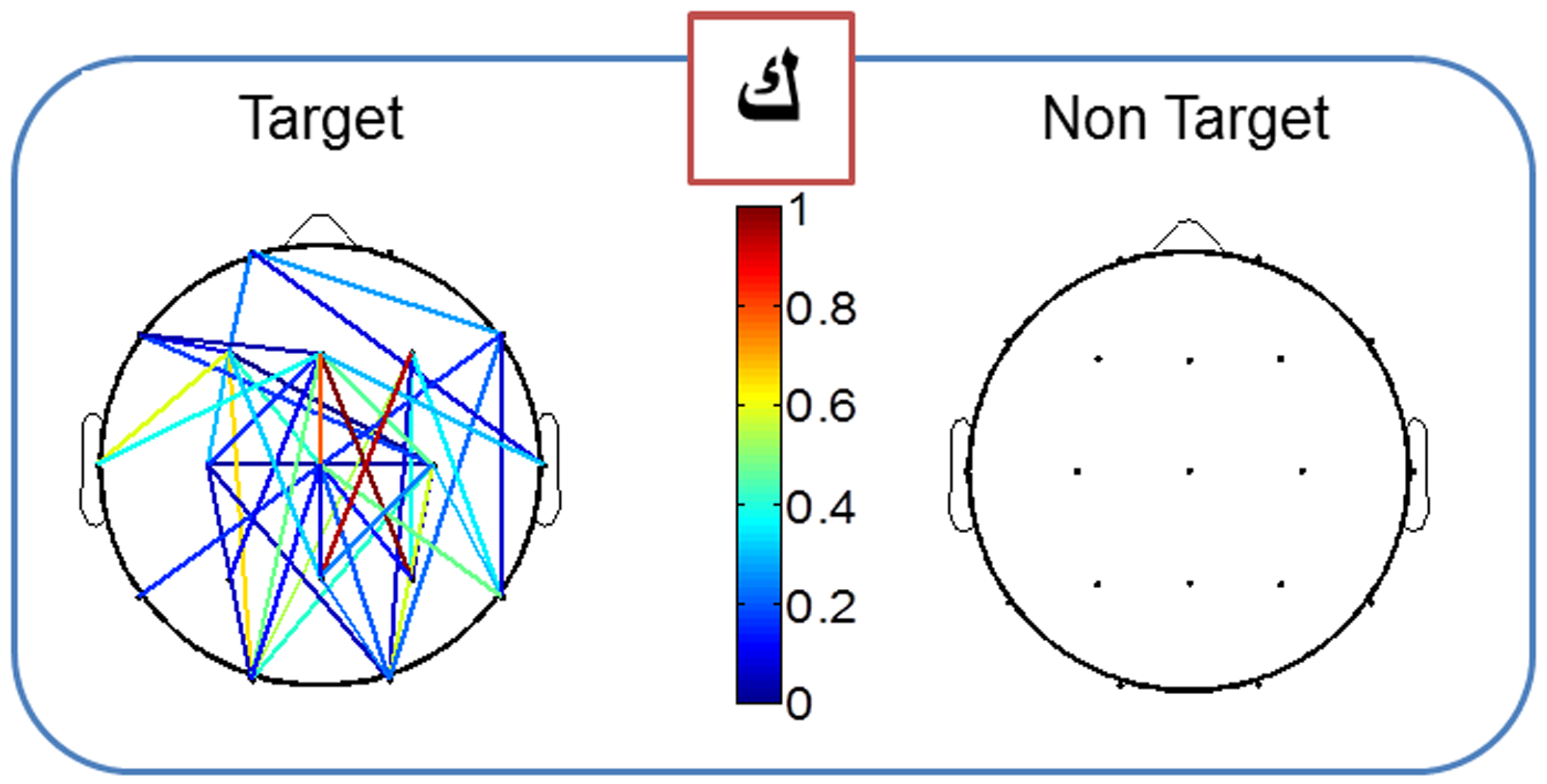

Supplement: S9 Fig — (TIF) [file pone.0146282.s009.tif]

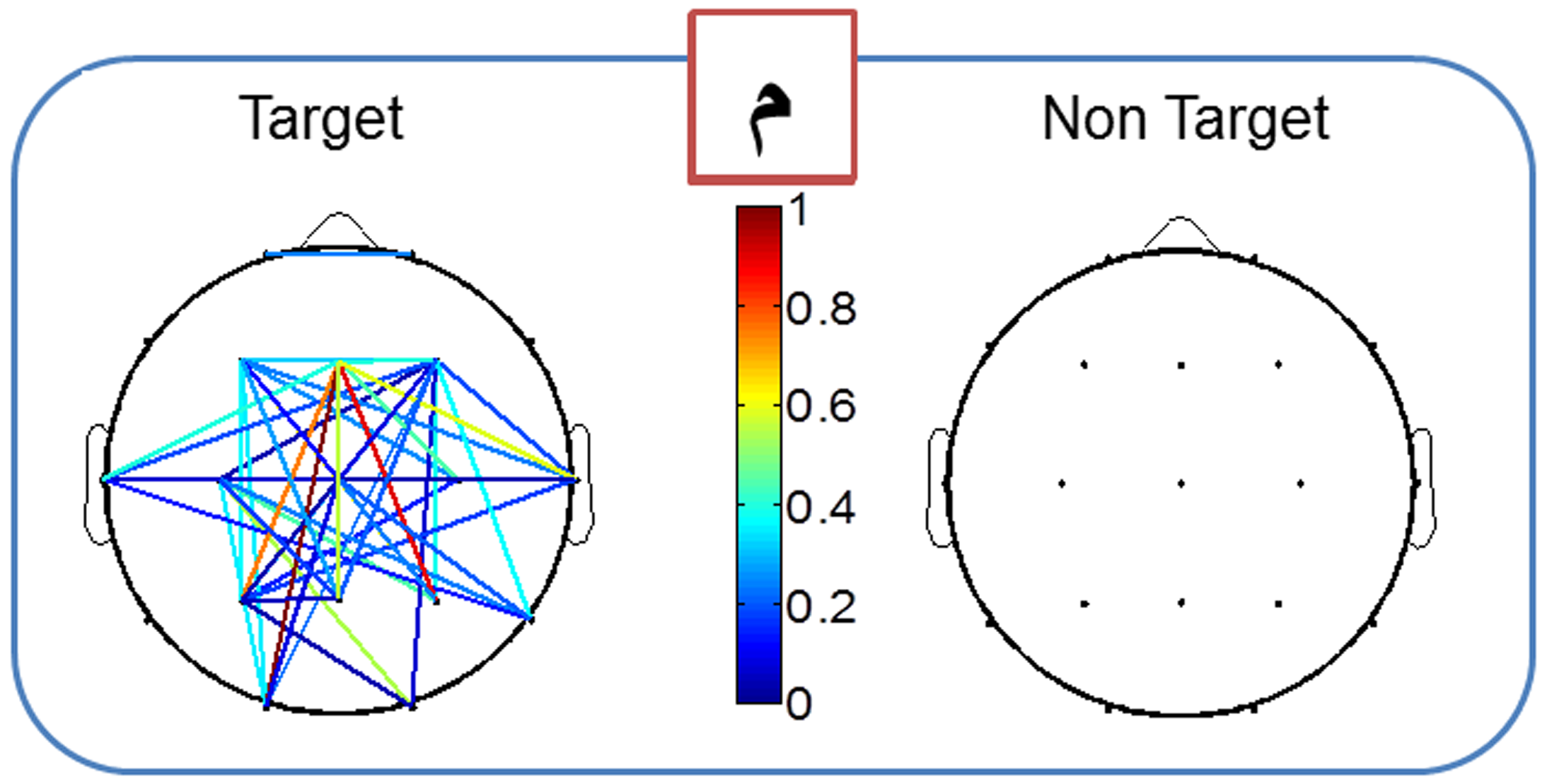

Supplement: S10 Fig — (TIF) [file pone.0146282.s010.tif]

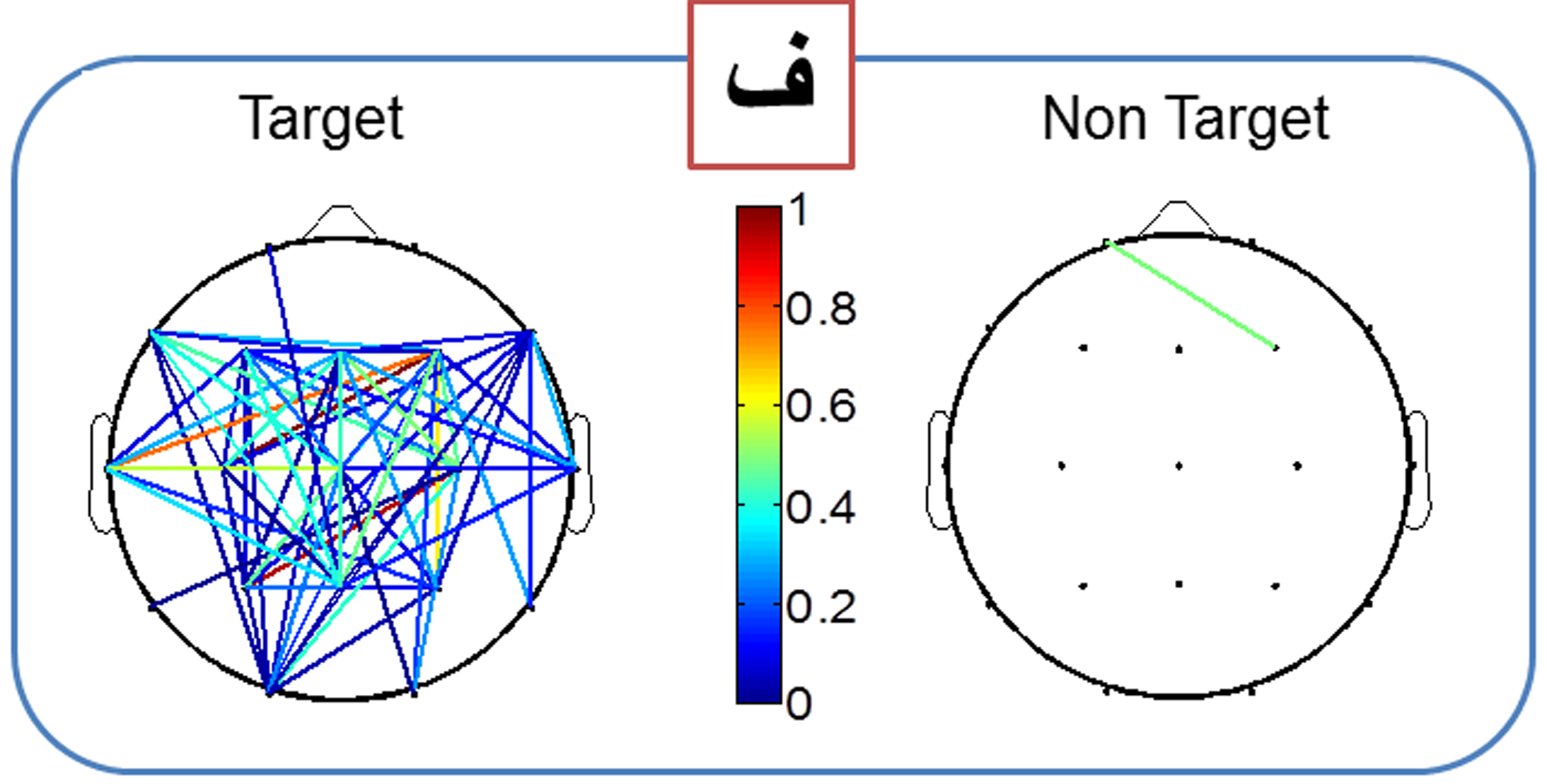

Supplement: S11 Fig — (TIF) [file pone.0146282.s011.tif]
